# The Value of Stress-Gated Blood Pool SPECT in Predicting Early Postoperative Period Complications in Ischemic Cardiomyopathy Patients: Focus on Mechanical Dyssynchrony

**DOI:** 10.3390/jcm12165328

**Published:** 2023-08-16

**Authors:** Vladimir V. Shipulin, Sergey Andreev, Kristina Kopeva, Vladimir M. Shipulin, Konstantin Zavadovsky

**Affiliations:** 1 Nuclear Department, Cardiology Research Institute, Tomsk National Research Medical Center, Russian Academy of Sciences, Tomsk 634012, Russia; shipartphoto@gmail.com (V.V.S.); konstzav@gmail.com (K.Z.); 2 Surgical Department, Cardiology Research Institute, Tomsk National Research Medical Center, Russian Academy of Sciences, Tomsk 634012, Russia; anselen@rambler.ru (S.A.); shipulin@cardio-tomsk.ru (V.M.S.); 3 Department of Myocardial Pathology, Cardiology Research Institute, Tomsk National Research Medical Center, Russian Academy of Sciences, Tomsk 634012, Russia

**Keywords:** ischemic cardiomyopathy, stress-gated blood pool SPECT, early postoperative period, prognosis, mechanical dyssynchrony

## Abstract

(1) Objective: The objective of this study was to assess the prognostic value of stress-gated blood pool SPECT (GBPS) estimates in patients with ischemic cardiomyopathy (ICM) in the early postoperative period. (2) Methods: A total of 57 patients (age 59.7 ± 6.6, 47 men) with ICM and LV ejection fraction (30 [27.5; 35]%) were enrolled in the study. Before surgical treatment, all patients underwent GBPS (rest–stress, dobutamine doses of 5/10/15 µg/kg/min). Stress-induced changes in left ventricular (LV) ejection fraction, peak ejection rate, volumes, and mechanical dyssynchrony (phase histogram standard deviation, phase entropy (PE), and phase histogram bandwidth) were estimated. Two-dimensional transthoracic echocardiography was performed baseline. Serum levels of NT-proBNP were analyzed with enzyme-linked immunoassay. (3) Results: After surgical treatment, patients were divided into two groups, one, with death, the need for an intra-aortic balloon pump (IABP) or/and inotropic support with a stay in the intensive care unit for more than two days and two, without complications in the early postoperative period (EPOP). Complicated EPOP (CEPOP) was observed in 17 (30%) patients (death—2, IABP—4, extra inotropic support in intensive care unit—11), and 40 patients had no complications (NCEPOP). GBPS showed differences in LV EDV (mL) (321 [268; 358] vs. 268 [242; 313], *p* = 0.02), LV ESV (mL) (242 [201; 282] vs. 196 [170; 230], *p* = 0.005), and stress-induced changes in PE (1 (−2; 3) vs. −2 (−4; 0), *p* = 0.02). Aortic cross-clamp time and stress-induced changes in PE between rest and dobutamine dose of 10 µg/kg/min were the only independent predictors of CEPOP. An increase in LV entropy ≥ 1 on the dobutamine dose of 10µg/kg/min in comparison to rest investigation showed AUC = 0.853 (sensitivity = 62%, specificity = 90%, PPV = 71%; NPV = 85%; *p* < 0.0001). Conclusion: Stress-induced changes in PE obtained during low-dose dobutamine GBPS are associated with a complicated course of the early postoperative period after surgical treatment for ICM.

## 1. Introduction

Surgical treatment for ischemic cardiomyopathy (ICM) remains a challenging task. These patients are at high risk of a complicated course of the early postoperative period [1]. Low cardiac output syndrome (LCOS) is one of the most common and dangerous complications after cardiac surgery, particularly in ICM patients. This condition is characterized by reduced oxygen delivery due to decreased systolic function [2]. It is strongly associated with increased morbidity and mortality in the short- and long-term postoperative period [3]. Patients with reduced LVEF develop LCOS more frequently than those with normal LVEF [4].

There are some data about preoperative and intraoperative predictors of LCOS [3,5]. The pathophysiology of this condition includes ischemic/reperfusion injury after coronary artery bypass grafting procedure with cardioplegic arrest, which can vary from temporary to persistent [3,5]. The severity of this disorder may be conditioned by the extent of preoperative myocardial systolic dysfunction, which can be assessed with nuclear modalities.

Phase analysis based on cardiac single photon emission computed tomography (SPECT) data, reflecting ventricular mechanical dyssynchrony (MD), is a very promising and rapidly developing tool due to its high reproducibility and almost complete automation [6,7,8]. This approach showed predictive capabilities in a number of diseases [9,10,11,12]. Despite the wide usage of myocardial perfusion imaging for this purpose, the feasibility of this approach is controversial in the presence of extent perfusion defects. Gated blood pool SPECT (GBPS) overcomes this disadvantage since the contractility analysis is based on blood pool contouring and does not depend on the presence and extent of myocardial perfusion defects. One of the latest trends in phase analysis is an investigation of stress-induced changes in phase histogram indices and their association with outcomes [13]. However, the vast majority of these studies have been performed with vasodilator stress, which is less informative for detecting wall motion abnormalities than stress tests with inotropic agents. Only a few studies have investigated stress-induced changes in MD caused by inotropic stimulation [13,14], including the use of GBPS [6,13]. Nevertheless, there are no data related to the predictive value of stress GBPS-derived LV MD changes and complicated EPOP in patients with ICM.

The objective of the present study was to determine the prognostic value of dobutamine-induced LV MD to predict LCOS and cardiac death in the early postoperative period after surgical treatment for ICM.

## 2. Methods

### 2.1. Patient and Study Population

A total of 77 patients scheduled for surgical treatments for ICM who underwent low dobutamine dose (LDD) gated blood pool SPECT (GBPS) were prospectively enrolled in the study between 2018 and 2020. The inclusion criteria were the following. Reduced LV systolic function (LVEF < 40%) and an increase in LV volumes using the 2-dimensional transthoracic echocardiography (TTE) data, history of myocardial infarction, more or equal to 75% stenosis of left main or ≥75% stenosis in the proximal left anterior descending artery, and/or stenosis of ≥75% of two or more epicardial vessels based on invasive coronary angiography results [1].

Patients who had contraindications to the LDD stress test, such as acute coronary syndrome, severe aortic valve stenosis, hypertrophic cardiomyopathy, hemodynamic instability, inflammatory myocardial disease, atrial fibrillation, severe hematological or neurological disorders, and patients who did not sign the informed consent were excluded from the study [15].

### 2.2. Study Design and Primary Endpoint

According to the research protocol, all patients underwent LDD GBPS within 3 days prior to surgery. In the early postoperative period (30 days after surgical treatment), the patients were divided into two groups: (1) patients with complications in the early postoperative period (CEPOP) and (2) those with no early postoperative complications (NCEPOP).

The primary endpoint of this study was a composite of LCOS (need for intra-aortic balloon pump (IABP) and/or inotropic support requiring more than 2 days in the intensive care unit and/or cardiac death within 30 days after surgery [16,17].

This research is part of the SciCoRIC study (ClinicalTrials.gov identifier: NCT04508608). The study was approved by the Local Ethical Committee and conformed to the Declaration of Helsinki on Human Research. Written informed consent was obtained from each patient after an explanation of the protocol, its aims, and potential risks.

### 2.3. GBPS Data Acquisition and Processing

The protocol of stress testing and GPBS data acquisition and processing have been described in detail in our previous article [6]. Briefly, after in vivo labeling of red blood cells with a ^99^mTc-pertechnetate dose of approximately 9 MBq/kg Corbett, et al. [17], patients were imaged in the supine position with the left arm placed over the head. An acquisition at rest was followed by dobutamine infusion at a dose of 5 µg/kg/min to achieve a stable response and then by consecutive infusions of 10 and 15 µg/kg/min with simultaneous GBPS data acquisition. Each stage lasted for 5 min. The 12-lead electrocardiogram was monitored continuously during the entire examination. Blood pressure was measured at baseline and repeated at each stage of the dobutamine infusion. The data were acquired with electrocardiogram-gating (16 frames/cycle; acceptance window of ±15%) for 5 min with a 20% energy window centered at 140 keV. No attenuation correction was used.

All examinations were performed using a dedicated cardiac CZT-SPECT gamma camera Discovery NM/CT 570c (GE Healthcare, Haifa, Israel). A syringe pump, Braun Perfusor Compact S (Braun, PA, USA), was used for the infusion of dobutamine.

Raw data were analyzed visually for motion and attenuation artifacts. Quality control was performed according to the European Association of Nuclear Medicine guidelines [17]. Images were reconstructed on a dedicated workstation (Xeleris 4.0110; GE Healthcare, Haifa, Israel) using maximum-penalized-likelihood iterative reconstruction (60 iterations; Green OSL Alpha 0.7; Green OSL Beta 0.3). The Myovation for Alcyone software v.6 (GE Healthcare, Haifa, Israel) was used for image reconstruction. The Butterworth post-processing filter (frequency 0.52; order 5) was applied to the reconstructed slices obtained in a 70 × 70 pixels matrix with 57 slices.

Reconstructed images were processed using the Quantitative Blood Pool SPECT 2009.0 (Cedars-Sinai Medical Center, Los Angeles, CA, USA) software. Ventricular contours, mitral, and tricuspid valves plane were adjusted manually when required. The count-based volumes method was used to calculate end-diastolic volume (EDV), end-systolic volume (ESV), as well as EF Corbett, et al. [17]. The following LV parameters were evaluated in further analysis: EDV (mL), ESV (mL), EF (%), and peak ejection rate (PER, expressed as EDV/s). The phase standard deviation (PSD), histogram bandwidth (HBW), and phase entropy (PE) were estimated as indices of mechanical dyssynchrony. Changes in GBPS parameters (delta, Δ) on each dobutamine dose were calculated as stress minus rest value.

### 2.4. Surgical Techniques

Preoperative preparation and intraoperative monitoring were standard. The surgical concept when performing surgical interventions strictly obeyed the “triple V” principle [18]. Surgical ventricular restoration was performed according to the Menicanti technique [19]. Mitral valve repair for ischemic mitral regurgitation in all cases was carried out using a Carpentier–Edwards rigid annuloplasty ring.

### 2.5. Echocardiography

Echocardiography was performed for all patients according to a standard protocol using an EPIQ device (Philips Ultrasound, Inc., Reedsville, PA, USA). The structures of the heart were visualized using B-scan according to the standard technique. All measurements were taken while patients were at rest and in the left lateral decubitus position and by following recommendations for cardiac chamber quantification by echocardiography in adults endorsed by the American Society of Echocardiography (ASE) and the European Association of Cardiovascular Imaging (EACVI) [20]. A single on-site specialist performed all echocardiographic examinations. Measurements included quantification of left ventricle (LV) internal dimensions in diastole (EDD, mm) and systole (ESD, mm) in parasternal long-axis view. LV ejection fraction (LVEF) was measured several times with the 2D biplane method according to the modified Simpson’s rule, and the average value was recorded [20].

### 2.6. Blood Sampling and Biochemical Analysis

Blood samples were obtained by venipuncture, and adequate centrifuged serum samples were stored at −26 °C with a single freeze–thaw cycle. Serum levels of NT-proBNP were analyzed with enzyme-linked immunoassay (Biomedica immunoassays, Vienna, Austria).

### 2.7. Statistical Analysis

Data were assessed for normal distribution using the Shapiro–Wilk test. Continuous variables were expressed as mean ± standard deviation (SD) or as median with interquartile range (IQR) (Q25 to Q75). Differences between independent groups were determined using the Mann–Whitney U test, and Wilcoxon signed-rank tests were performed for unpaired and paired data, respectively. The χ^2^ or Fisher’s exact tests were used for categorical variables. Friedman ANOVA was used to reveal differences in GBPS parameters measured during the entire study, at rest and for 10 and 15 µg/kg/min of dobutamine. When the difference was statistically significant, a post-hoc paired comparison was applied using the Wilcoxon signed rank test with Bonferroni correction for multiple comparisons. Each statistical test was 2-sided, and a *p*-value of <0.05 was considered statistically significant.

A logistic regression analysis was performed to assess the relationship between parameters and the course of EPOP. Independent predictors of CEPOP were evaluated using forward-stepwise logistic regression analysis with an entry criterion of *p* < 0.05 and a removal criterion of *p* > 0.1.

Receiver-operating characteristic (ROC) curves were analyzed to determine the optimal cut-off values and diagnostic performance (sensitivity, specificity, positive (PPV), and negative (NPV) predictive values) to predict CEPOP. Statistical analyses were performed using STATISTICA 10.0 (StatSoft Inc., Tusla, OK, USA) and MedCalc 17.4 (MedCalc Software, Mariakerke, Belgium).

## 3. Results

**Figure 1:**
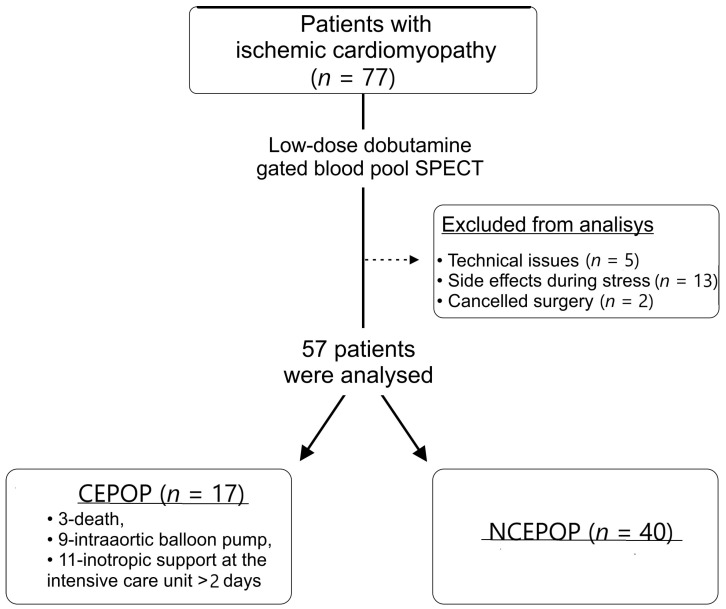
Study flowchart. CEPOP—complicated early postoperative period; NCEPOP—uncomplicated early postoperative period.

Among all 77 recruited patients, 5 had technical issues during the GBPS acquisition, 13 patients had side effects during stress (chest pain (*n* = 6), excessive hypertensive response (*n* = 6), and heart rhythm disturbances (*n* = 4) resulting in test termination and 2 patients had surgery canceled. As a result, 57 ICM patients (age 59.7 ± 6.5 years, 47 men) were included in the final analysis (Figure 1). All patients underwent coronary bypass grafting, 25 (44%) received LV reconstruction, and 12 (21%) received mitral valve repair.

A total of 17 (30%) patients who had complications in the early postoperative period (death—3, IABP—9, the need for inotropic support in the intensive care unit >2 days—11) formed the CEPOP group, whereas 40 (70%) had no complications and formed the NCEPOP group. The clinical characteristics of the patient sample are presented in Table 1. The CEPOP group was characterized by a higher incidence of dyslipidemia (*p* = 0.02) and lower values of ESV (*p* = 0.01) as well as longer aortic cross-clamp (XCL) time (*p* = 0.04), as compared to NCEPOP patients (Table 1). All patients received optimal medical therapy according to current clinical recommendations. The groups did not differ significantly in the frequency of distribution of prescribed drugs.

According to the rest GBPS results, CEPOP patients had significantly higher values of EDV (*p* = 0.007) and ESV (*p* = 0.006) in comparison to NCEPOP (Table 2).

**Figure 2:**
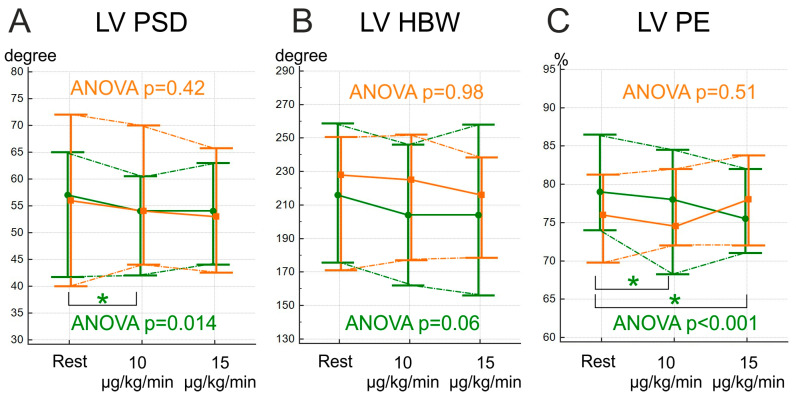
Stress-induced changes in left ventricle mechanical dyssynchrony indices during stress-test. The values are expressed as median and IQR (25–75). (**A**)—phase standard deviation (PSD); (**B**)—histogram bandwidth (HBW); (**C**)–phase entropy (PE); Orange lines and data—CEPOP; Green lines and data—NCEPOP; LV—left ventricle; Data are expressed in median and IQR (25;75). *—*p* < 0.016.

**Figure 3:**
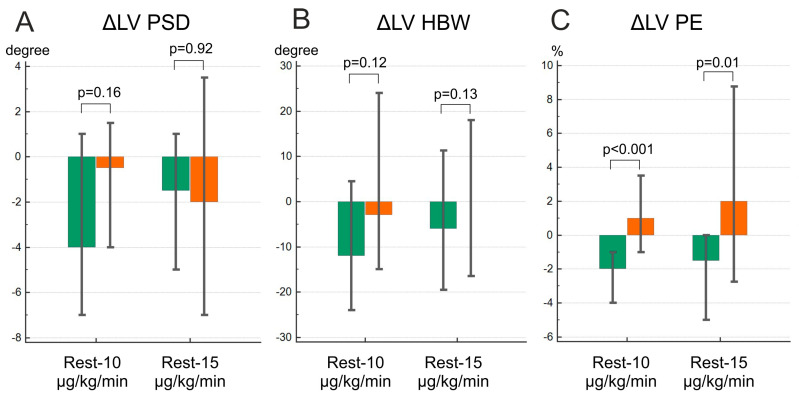
Deltas of dyssynchrony parameter changes. (**A**)—phase standard deviation (PSD); (**B**)—histogram bandwidth (HBW); (**C**)—phase entropy (PE); Green bar—NCEPOP; Orange bar—CEPOP; LV—left ventricle. Data are expressed in median and IQR (25;75).

Based on the Friedman ANOVA test, significant changes during the dobutamine stress test were observed only in the NCEPOP group (PSD on the dobutamine dose of 10 µg/kg/min and PE on both dobutamine doses compared to the study at rest) (Figure 2). Nevertheless, PE in the CEPOP group showed an upward trend, more pronounced at the dobutamine dose of 15 µg/kg/min, while other MD indices demonstrated predominantly a decrease in or absence of changes. The comparison of deltas of MD between the groups revealed that only ∆PE differed significantly (*p* < 0.001 at the dobutamine dose of 10 µg/kg/min and *p* = 0.01 at the dobutamine dose of 15 µg/kg/min) (Figure 3). The GBPS values on each dobutamine dose are available in Supplementary Materials (Table S1).

The result of univariate and multivariate logistic regression analyses are shown in Table 3. In the multivariate analysis, the stress-induced changes in PE at the dose of 10 µg/kg/min, along with the XCL time, were the only independent predictors of the composite endpoint (OR = 1.67; CI 1.13–2.49, *p* < 0.001 and OR = 1.02, CI 1.001–1.05; *p* < 0.001, respectively).

**Figure 4:**
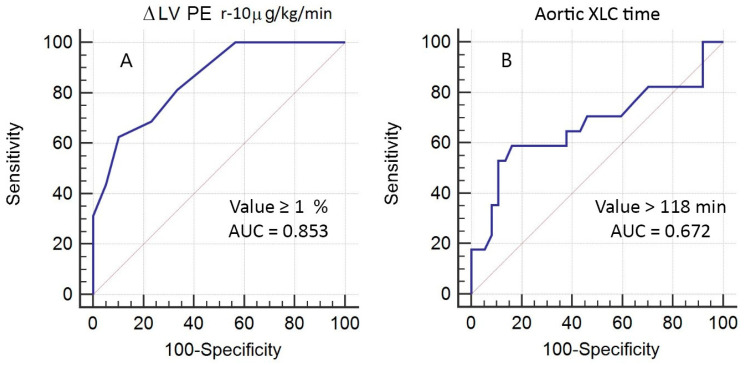
ROC analysis of independent predictors of CEPOP after surgical treatment of ICM. (**A**)—stress-induced changes (Δ) of left ventricular (LV) phase entropy (PE) between rest (r) and dobutamine dose of 10 µg/kg/min; (**B**)—aortic cross-clamp (XCL) time.

The ROC analysis revealed that ∆PE at the dobutamine dosage of 10 µg/kg/min ≥1% has area under curve (AUC) = 0.853 (sensitivity = 62%, specificity = 90%, PPV = 71%; NPV = 85%, *p* < 0.0001) and XCL time > 118 min showed AUC = 0.672 (sensitivity = 59%, specificity = 84%, PPV = 62%; NPV = 82%, *p* = 0.05) in the prediction of complicated EPOP (Figure 4).

## 4. Discussion

The main findings of this study are the following. (1) An increase in LV PE during the dobutamine stress test is associated with complicated EPOP in patients after surgical treatment of ICM and comprises LCOS and cardiac death. (2) A dobutamine dose of 10 µg/kg/min is sufficient to identify patients at high risk of LCOS and cardiac death. To the best of our knowledge, this is the first study that reveals the association between stress-induced changes in mechanical dyssynchrony obtained from GBPS with a CZT gamma camera and adverse EPOP in patients with ICM.

### 4.1. The Value of Mechanical Dyssynchrony

Although MD can be assessed with multiple radiation-free imaging techniques, such as TTE and cardiac magnetic resonance, there are some crucial advantages of nuclear modalities in this area. SPECT has the lowest inta- and inter-observer variability; acquisition is relatively fast and provides the operator with information about perfusion along with contractile function [6,7,14]. Additionally, there are no strict contraindications for radiopharmaceuticals [15].

Several previous studies showed a prognostic value of MD revealed by nuclear modalities in different cardiac pathologies [10,11,12,21,22,23], including patients with severe heart failure [9,11,24,25,26]. However, only a few of them were focused on PE as a key dyssynchrony variable [12,24,27,28].

Interest in the study of PE is growing rapidly. This is a rather “young” parameter, reflecting the degree of phase histogram disorder [29,30]. The nature of PE is not yet completely clear; it is known that entropy may be influenced by LV volume, EF, age, and severity of heart failure [29,30]. On the other hand, PE is less influenced by outliers and the shape of the histogram, allowing considering this parameter as more robust as compared to PSD and HBW [29,30]. Moreover, in patients with ICM, PE showed higher repeatability as compared to PSD and HBW [14]. Interestingly, there was reported a reverse correlation between a degree of LV PE (by rest MPI) and the level of SERCA2a mRNA expression [24], which mediates the contraction of cardiomyocytes and the re-entry of Ca^2+^ from the cytoplasm into the sarcoplasmic reticulum [31].

The prognostic value of PE at rest has been shown previously regarding several specific groups of patients. In patients with complete left bundle branch block, with LVEF = 49.2% and normal LV volumes, PE ≥ 79% was associated with adverse cardiac events over a 2-year follow-up (risk ratio 7.58; 95% CI 2.39–24.08; *p* < 0.001) [28]. In ICM patients, MPI SPECT-derived PE > 59% increased the risk of cardiac death by a factor of 4.25 (*p* = 0.039), while neither MD indices obtained by positron emission computed tomography nor QRS duration showed any predictive value [27]. These discrepancies may be explained by significant differences in MD parameters determined by various methods [32] and different natures of electrical and mechanical dyssynchrony [33], respectively. In DCM patients with QRS < 120 ms, PE ≥ 61% was a predictor of adverse cardiac events (risk ratio 5.7; CI 95% 1.02–108.32; *p* = 0.047) [24]. However, according to our results among patients with ICM who underwent cardiac surgery, MD indices at rest did not reveal any prognostic value in terms of complicated EPOP.

### 4.2. The Value of Stress-Induced Mechanical Dyssynchrony

MD can be underestimated at rest. Stress tests are usually used to reveal MD, especially in CAD [16]. However, existing data are mainly based on post-stress measurements taken at least 15 min (more often 45–60 min) after the stress test [12,34,35]. Contractility disturbances observed on post-stress investigations (usually expressed as increased MD indices) may reflect myocardial stunning [34,36]. Nevertheless, stunning is a time-dependent phenomenon and may disappear by the time perfusion imaging is performed [37,38].

Only a few investigations acquired SPECT data directly during the stress test [6,39,40]. In the experimental study by Salimian S. et al. [40] on the canine model, all SPECT-MPI-derived dyssynchrony parameters demonstrated a falling trend with an increase in the dobutamine level (suggesting more synchronous mechanical activation during stress). These results were later confirmed in the clinical study based on ICM patients [14]. However, such an approach (based on SPECT-MPI) can lead to mistakes in the evaluation of MD in the presence of extent perfusion defects due to low count levels and difficulties in delineation of the myocardial contour [25,41]. This last disadvantage of SPECT-MPI can be overcome using GBPS, which visualizes the blood pool and does not depend on myocardial wall contouring. The former study, which performed GBPS with a dobutamine stress test based on tachycardia-induced DCM dogs, confirmed the initial data about the presence of “synchrony reserve”, a decrease in MD during a stress test [39], while the latter clinical study with ICM patients demonstrates an association between a drop of LVEF and an increase in PE during a stress test. All of these studies have shown that PE more accurately reflects dobutamine-induced changes in myocardial contractility compared to other MD parameters. Nevertheless, none of these studies examined the prognostic value of stress-induced changes in PE.

The current work provides new insight into the prognostic value of MD. This study revealed that the increase in PE at a dobutamine dose of 10 µg/kg/min, measured before surgery, was associated with complicated EPOP (LCOS, cardiac death within 30 days after surgery) after surgical treatment of ICM. The exact mechanism of association between PE and adverse EPOP is difficult to determine. Most interventions implying cardiopulmonary bypass with cardioplegic arrest or XCL could lead to myocardial dysfunction due to ischemic/reperfusion injury [5]. It is known that stress-induced increased MD is associated with ischemia and myocardial stunning [34,36,37,42]. These facts suggest that a stress-induced increase in PE may reflect the exhaustion of myocardial contractile potential and intolerance to ischemia, which leads to a decrease in LV contractile function after surgical intervention.

### 4.3. The Value of Dobutamine Dosage

This study revealed that a dobutamine dose of 10 µg/kg/min is sufficient for the evaluation of patients at high risk after surgical treatment for ICM. The existing differences in PE stress-induced changes between groups appeared at this dose, while further dose increases did not cause any significant changes. In our previous study, we showed that a dobutamine dosage of 10 µg/kg/min is enough to evaluate stress-induced changes in LVMD in ICM patients [6], which is in line with previous studies [39,40]. Although an increase in PE at the dobutamine dose of 15 µg/kg/min in the CEPOP group was observed, stress-induced changes in PE at the 10 µg/kg/min dose showed a stronger association with EPOP complications. Low dobutamine dosages (2.5–10 µg/kg/min) are usually used to assess myocardial contractile reserve, whereas higher doses are used to assess disorders caused by ischemia but increase the risk of side effects [16,43]. MD parameters are more sensitive in the detection of coronary artery disease as compared to perfusion and transient ischemic dilatation [44], so it might detect changes caused by ischemia at a lower level of stress.

## 5. Conclusions

Stress-induced changes in LV PE by preoperative low-dose dobutamine GBPS are associated with the complicated course of the early postoperative period after surgical treatment for ICM.

The rise in PE values at a dobutamine dose of 10 μg/kg/min is the independent predictor of the adverse early postoperative period, while a further increase in the dose had no additional prognostic value. Further large-scale clinical studies are needed to evaluate the potential clinical significance of this approach in patients with severe heart failure.

## 6. Limitations

This is a single-center study with a relatively small number of patients; nevertheless, it demonstrates the feasibility and potential of stress GBPS to predict early postoperative complications after surgical treatment of ICM. The absence of a control group not undergoing CABG limits the clinical translation of the study findings. In the present study, we assessed LV contractile function based on 5 min data acquisition. While there may be some averaging of the contractile function during this acquisition time, its effect, particularly at 10 and 15 μg/kg/min of dobutamine, can be considered insignificant because of dobutamine saturation at the dose of 5µg/kg/min. We used global parameters of LV mechanical synchrony and did not take into consideration the location of the infarct zone and the regional parameters.

## Figures and Tables

**Table 1:** Baseline characteristics of included patients.

Parameter	Total (*n* = 57)	CEPOP (*n* = 17)	NCEPOP (*n* = 40)	*p*-Value
Age (years)	59.7 ± 6.5	61 ± 6.1	58 ± 7.4	0.2
Dyslipidemia, *n* (%)	41 (72%)	15 (88%)	26 (65%)	0.02 *
Hypertension, *n* (%)	50 (88%)	16 (94%)	34 (85%)	0.21
Type 2 DM, *n* (%)	13 (23%)	3 (17%)	10 (25%)	0.41
NYHA class, n (%):				
II	31 (55%)	7 (41%)	24 (60%)	0.6
III	28 (45%)	10 (59%)	16 (40%)	
LV EF (%) **, *n* (%)	30 (27.5; 35)	30 (25; 31)	32 (29; 35)	0.09
LV EDV (mL) **, *n* (%)	210 (187; 235)	229 (199; 273)	208 (187; 227)	0.06
LV ESV (mL) **, *n* (%)	142 (129; 166)	158 (136; 198)	136 (128; 157)	0.01 *
The number of CA with stenosis > 75%, *n* (%):				
1	7 (12%)	1 (6%)	6 (15%)	
2	9 (16%)	2 (12%)	7 (18%)	0.16
3	41 (72%)	14 (82%)	27 (67%)	
EuroSCORE 2 (%)	4.3 (2.5; 5.7)	4.6 (4;1; 6.2)	3.5 (2.2; 5.2)	0.1
LV reconstruction	25 (44%)	7 (41%)	18 (45%)	0.77
MV repair	12 (21%)	6 (35%)	6 (16%)	0.15
CPB time (min)	143 (110; 178)	172 (149; 190)	124 (110; 173)	0.06
Aortic XCL time (min)	97 (74; 123)	125 (76; 133)	83 (72; 105)	0.04 *
eGFR (mL/min/1.73 m^2^)	56 (45; 72)	54.6 (42.3; 76)	55.5 (44.6; 78)	0.08
NT-proBNP, pg/mL	678 (458; 882)	638 (424; 879)	515 (405; 998)	0.6

Note. CEPOP—complicated early postoperative period; NCEPOP—uncomplicated early postoperative period; NYHA—functional class of heart failure according to the New York Heart Association; LV—left vertical; EF—ejection fraction; EDV—end-diastolic volume; ESV—end-systolic volume; CA—coronary arteries; EuroSCORE 2—European System for Cardiac Operative Risk Evaluation; MV—mitral valve; CPB—cardio-pulmonary bypass; XCL—aortic cross-clamp; eGFR—estimated glomerular filtration rate (CKD-EPI); NT-proBNP—N-terminal pro-B-type natriuretic peptide; *—statistically significant; **—data obtained from TTE.

**Table 2:** Left ventricular gated blood pool SPECT parameters.

Parameter	CEPOP (*n* = 17)	NCEPOP (*n* = 40)	*p*-Value
EDV rest (mL)	323 (281; 373)	265 (242; 306)	0.007 *
ESV rest (mL)	242 (298; 293)	198 (170; 232)	0.006 *
EF rest (%)	28 (18; 31)	28 (23;32)	0.33
PER rest (EDV/s)	−1.18 (−1.45; −0.7)	−1.27 (−1.48; −1.0)	0.24
PSD rest (degree)	56 (40; 72)	57 (41; 65)	0.75
HBW rest (degree)	228 (171; 250)	216 (175; 258)	0.81
PE rest (%)	76 (70; 81)	79 (74; 86.5)	0.35
Δ EF rest-10 µg/kg/min (%)	2.5 (1.5; 6)	3 (−1; 7)	0.97
Δ EF rest-15 µg/kg/min (%)	4 (1.2; 9.7)	4 (−0.7; 8)	0.74
Δ PER rest-10 µg/kg/min (%)	0.29 (−0.02; 0.44)	0.18 (−0.03; 0.44)	0.79
Δ PER rest-15 µg/kg/min (%)	0.32 (0.118; 0.55)	0.22 (0.04; 0.46)	0.64
Δ PSD rest-10 µg/kg/min (%)	−0.5 (−4; 1.5)	−4 (−7; 1)	0.16
Δ PSD rest-15 µg/kg/min (%)	−2 (−7; 3.5)	−1.5 (−5; 1)	0.92
Δ HBW rest-10 µg/kg/min (%)	−3 (−15; 24)	−12 (−24; 4.5)	0.12
Δ HBW rest-15 µg/kg/min (%)	0 (−16.5; 18)	−6 (19.5; 11.25)	0.13
Δ PE rest-10 µg/kg/min (%)	1 (−1; −3.5)	−2 (−4; −1)	<0.001 *
Δ PE rest-15 µg/kg/min (%)	2 (−2.7; 8.7)	−1.5 (−5; 0)	0.01 *

Note. EDV—end-diastolic volume; ESV—end-systolic volume; EF—ejection fraction; PER—peak ejection rate; PSD—phase standard deviation; HBW—histogram bandwidth; PE—phase entropy; Δ—stress-induces changes; CEPOP—complicated early postoperative period; NCEPOP—uncomplicated early postoperative period; *—statistically significant.

**Table 3:** Univariate and multivariate logistic regression analyses of parameters predicting adverse early postoperative period in patients with ICM.

Parameter	OR	CI (95%)	*p*-Value
Univariate logistic regression			
Dyslipidemia	9.75	1.16–81.53	0.007
LV ESV (mL) *	1.01	1.002–1.03	0.01
Aortic XCL time (min)	1.02	1.002–1.04	0.01
LV EDV (mL) **	1.01	1.002–1.02	0.005
LV ESV (mL) **	1.01	1.003–1.02	0.003
∆ PE rest-10 µg/kg/min (%)	1.85	1.27–2.69	<0.001
∆ PE rest-15 µg/kg/min (%)	1.24	1.06–1.44	<0.001
Multivariate logistic regression			
Aortic XCL time (min)	1.02	1.001–1.05	<0.001
∆ PE rest-10 µg/kg/min (%)	1.67	1.13–2.49	<0.001

Note. EDV—end-diastolic volume; ESV—end-systolic volume; PE—phase entropy; Δ—stress-induces changes; *—statistically significant; **—data obtained from TTE.

## Data Availability

The data presented in this study are available on request from the corresponding author. The data are not publicly available due to privacy.

## Supplementary Materials

The following supporting information can be downloaded at: https://www.mdpi.com/article/10.3390/jcm12165328/s1, Table S1. Left ventricular gated blood pool SPECT parameters at rest and on each dobutamine dose.
