# Supplementary material for: The Value of Stress-Gated Blood Pool SPECT in Predicting Early Postoperative Period Complications in Ischemic Cardiomyopathy Patients: Focus on Mechanical Dyssynchrony
Source: J Clin Med. 2023 Aug 16;12(16):5328. doi: 10.3390/jcm12165328 (PMC10455889; doi:10.3390/jcm12165328)
Supplement: Supplementary file 1 [file jcm-12-05328-s001.zip › jcm-2499286-supplementary.pdf]

Table S1. Left ventricular gated blood pool SPECT parameters at rest and on each dobutamine dose.

	CEPOP		NCPOP	
	Median	IQR	Median	IQR
EF rest (%)	28	18–31	28	23–32
EF 10 µg/kg/min (%)	29	23–33	31	28–36
EF 15 µg/kg/min (%)	32	23–34	33	26–35
EDV rest (ml)	323	281–373	265	242–306
EDV 10 µg/kg/min (ml)	334	260–368	260	221–296
EDV 15 µg/kg/min (ml)	338	272–397	261	232–310
ESV rest (ml)	242	198–293	198	170–232
ESV 10 µg/kg/min (ml)	224	177–281	176	146–215
ESV 15 µg/kg/min (ml)	220	184–300	174	155–224
PER rest (EDV/s)	−1.18	−1.45–−0.70	−1.27	−1.48–−1.00
PER 10 µg/kg/min (EDV/s)	−1.39	−1.53–−1.27	−1.39	−1.66–−1.26
PER 15 µg/kg/min (EDV/s)	−1.59	−1.75–−1.13	−1.42	−1.73–−1.33
PSD rest (degree)	56	40–72	57	41–65
PSD 10 µg/kg/min (degree)	54	44–70	54	42–60
PSD 15 µg/kg/min (degree)	53	42–65	54	44–63
HBW rest (degree)	228	171–250	216	175–258
HBW 10 µg/kg/min (degree)	225	177–252	204	162–246
HBW 15 µg/kg/min (degree)	216	178–238	204	156–258
Entropy rest (%)	76	69–81	79	74–86.5
Entropy 10 µg/kg/min (%)	74	72–82	78	68–84
Entropy 15 µg/kg/min (%)	78	72–83	75	71–82

EF – ejection fraction; EDV – end diastolic volume; ESV – end-systolic volume; PER – peak ejection rate; PSD – phase standard deviation; HBW – histogram bandwidth; Δ – stress-induces changes; CEPOP – complicated early postoperative period; NCEPOP – uncomplicated early postoperative period;
